# Structures of the *T. brucei* kRNA editing factor MRB1590 reveal unique RNA-binding pore motif contained within an ABC-ATPase fold

**DOI:** 10.1093/nar/gkv647

**Published:** 2015-06-27

**Authors:** Porsha L. R. Shaw, Natalie M. McAdams, Michael A. Hast, Michelle L. Ammerman, Laurie K. Read, Maria A. Schumacher

**Affiliations:** 1 Department of Biochemistry, Duke University School of Medicine, Durham, NC 27710, USA; 2 Department of Microbiology and Immunology, University at Buffalo School of Medicine, Buffalo, NY, USA

## Abstract

Kinetoplastid RNA (kRNA) editing is a process that creates translatable mitochondrial mRNA transcripts from cryptogene encoded RNAs and is unique for kinetoplastids, such as *Trypanosoma brucei*. In addition to the catalytic 20S editosome, multiple accessory proteins are required for this conversion. Recently, the multiprotein mitochondrial RNA binding complex 1 (MRB1) has emerged as a key player in this process. MRB1 consists of six core proteins but makes dynamic interactions with additional accessory proteins. Here we describe the characterization of one such factor, the 72 kDa MRB1590 protein. *In vivo* experiments indicate a role for MRB1590 in editing mitochondrial mRNA transcripts, in particular the transcript encoding the ATP synthase subunit 6 (A6). Structural studies show that MRB1590 is dimeric and contains a central ABC-ATPase fold embedded between novel N- and C-terminal regions. The N-terminal domains combine to create a basic pore and biochemical studies indicate residues in this region participate in RNA binding. Structures capturing distinct MRB1590 conformations reveal that the RNA binding pore adopts closed and open states, with the latter able to accommodate RNA. Based on these findings, implications for MRB1590 function are discussed.

## INTRODUCTION

Kinetoplastid parasitic protozoa, which include *Trypanosoma brucei, Trypanosoma cruzi* and *Leishmania* spp., are the causative agents of life threatening afflictions that include African sleeping sickness, chagas disease and leishmaniasis (1). Kinetoplastids are named for their unique mitochondrial DNA network, known as the kinetoplast, which is a dense catenated structure comprised of several DNA maxicircles and thousands of minicircles (1). Kinetoplastid protozoa employ a number of unusual biological processes that have been proposed as specific targets for drug development. Perhaps the most remarkable of these processes is kinetoplastid RNA editing (kRNA editing) (2–7). kRNA editing takes place in the mitochondria and involves the specific insertion and/or deletion of uridine nucleotides. This process is required to generate translatable mRNAs from otherwise nonfunctional cryptogene derived mRNAs and is directed by small RNAs called guide RNAs (gRNAs), which are primarily encoded on kinetoplastid minicircle DNA.

In *T. brucei* 12 of the 18 mRNA transcripts require editing to produce functional mRNAs (2–7). However, the extent of editing varies. Some, so-called minimally edited transcripts, require the insertion and deletion of only a few uridines, while pan-edited transcripts necessitate the insertion and deletion of hundreds of uridine nucleotides to create functional mRNA transcripts (2–10). gRNAs carry the information to mediate this process. Although sequentially distinct, gRNAs share a conserved tertiary structure comprised of three regions (11). The gRNA 5′-end contains a stem-loop with a sequence that is complementary to the transcript to be edited, following this ‘anchor sequence’ is a second stem-loop that harbors the information for how many uridines to be added or deleted. The 3′-end of the gRNA contains a string of 5–15 uridines that provides stability to the mRNA-gRNA interaction. kRNA editing proceeds in the 3′-5′ direction, whereby the editing of one region of the transcript provides a new site for the next round of editing (2–10).

The enzymes that catalyze editing form a stable complex called the 20S editosome (12–14). This multiprotein complex consists of endonuclease, exonuclease, uridyltransferase and ligase activities. Three 20S editosomes have been identified and differ in the endonuclease that is present. Interestingly, it has been suggested that these different editosome forms may be employed in the editing of specific transcripts (12). Data show that in addition to the 20S editosome, multiple accessory factors are required for editing (15–27). Many of these proteins show no sequence homology to any known protein. Hence, how they function at the molecular level has been largely unclear. To date, only two kRNA editing accessory factors have been characterized at the atomic level, the MRP1/MRP2 complex and p22 (16,17). MRP1/MRP2 acts as an RNA matchmaker by melting out the secondary structure present in the first stem-loop or anchor sequence of gRNAs to facilitate their interaction with pre-edited mRNAs. Crystal structures of MRP1/MRP2 showed that it forms a heterotetramer that belongs to the Whirly family of nucleic acid binding proteins. A structure of MRP1/MRP2 with bound gRNA revealed that it binds nonspecifically to gRNAs such that the bases are exposed on the molecular surface for interaction with the mRNA (15,16). While less understood, the p22 protein forms a trimer and displays structural homology to the eukaryotic p32 protein, which functions in RNA splicing. However, p22 does not bind RNA and *in vivo* experiments revealed that it is necessary for editing the transcript encoding cytochrome c oxidase subunit II (COXII). These studies suggest that p22 functions as an adaptor by linking the editosome to the kRNA editing protein, TbRGG2 (17).

More recently, a large multiprotein complex called, the mitochondrial RNA binding complex 1 (MRB1), was discovered and shown to be essential for kRNA editing (18–24). MRB1 was originally identified through immunoaffinity experiments using the gRNA-associated proteins 1 and 2 (GAP1 and GAP2), which are required for gRNA stability in kinetoplastid mitochondria (19–20,25). These studies showed that a core MRB1 complex consists of GAP1/GAP2, and four or five other components, MRB3010, MRB5390, MRB8620, MRB11870 and MRB0880 (22). MRB3010 was found to be necessary for viability of both the procyclic and bloodstream forms of *T. brucei;* when MRB3010 is knocked down, kRNA editing is largely abrogated. Data revealed that MRB3010 is essential because it appears to play a central role in the initiation of kRNA editing (21). MRB11870 down regulation leads to an increase in pre-edited mRNA transcripts and studies showed that this protein is required for proper formation of the MRB1 complex by its interaction with GAP1/GAP2 (23). Subsequent work on MRB1 has implicated an array of possible functions for this complex, making it difficult to pinpoint its specific *in vivo* role(s) in kRNA editing. However, the current model posits two key functions for this complex. The first is in synchronizing multiple rounds of editing by the editosome by somehow mediating the exchange of the many gRNAs needed to edit pan-edited mRNAs. A second important function of the MRB1 complex is in coordinating kRNA editing with other RNA processing events (24,26). Indeed, it has been unclear how the pre-edited mRNA is transferred from the transcription to the editing machinery as well as how the mRNA generated by kRNA editing is processed for stability and then handed off to the ribosome. The functions of MRB1 appear to be involved in at least some of these processes and hence explain some of the long-standing questions in kRNA editing biology.

A key aspect of MRB1 function, however, that has made it difficult to study is its dynamic nature. Specifically, in addition to core proteins, a number of other factors have been shown to associate with MRB1 during editing. One such protein, MRB1590, was originally identified in MRB1 complexes immunoprecipitated or tandem affinity purified with GAP1/GAP2 (21). MRB1590 was also observed in pull-downs of the MRB1 components, MRB10130, MRB4140 and MRB8170 (22,27). However, this protein was absent from other MRB1 purifications, suggesting that its association with the MRB1 complex is dynamic (19–21). Here we carry out a detailed structure-function study on the MRB1590 protein. *In vivo* experiments suggest that MRB1590 effects editing of several mitochondrial mRNA transcripts, in particular that encoding subunit 6 of ATPase synthase (A6). Structural studies show that MRB1590 consists of three domains. These include novel N-terminal and C-terminal domains, which encompass a central region that contains an ATPase ABC fold. We show that residues in the basic N-terminal domain are involved in RNA binding and crystal structures capturing open and closed states of MRB1590 suggest possible mechanisms for how this protein may participate in RNA editing processes.

## MATERIALS AND METHODS

### Generation of procyclic form *T. brucei* cell lines

To generate MRB1590 RNAi cells, a fragment of the MRB1590 open reading frame from nucleotides 1299–2002 was amplified using PCR and cloned into the BamHI and ClaI sites of p2T7–177 (28) that had been modified to contain the puromycin resistance gene. Transformants were generated as previously described (28), including selection with 1 μg/ml puromycin. Repression of MRB1590 was induced by adding 2.5 μg/ml tetracycline to cell cultures.

For integration of the tandem affinity purification PTP tag into the endogenous MRB1590 locus, primers MRB1590 5′ ApaI (5′-GAGGGCCCCCATTACGCCTTTCGTGGAGCG-3′) and MRB1590 3′ NotI (5′-GAGCGGCCGCCTTTTCGCAGTAACGGTCCGGAG-3′), from nucleotides 1299–2002 of the MRB1590 open reading frame, were used to amplify the 3′ end of MRB1590 and introduce it into the ApaI-NotI restriction sites of pC-PTP PURO (28). pC-PTP-MRB1590 was then restricted at the unique XagI site within MRB1590 and transfected into procyclic form *T. brucei* 29–13 cells. Cells were selected with 1 μg/ml puromycin and cloned by limiting dilution.

### Quantitative real time PCR

Total RNA was isolated from uninduced and tetracycline-induced MRB1590 RNAi cells on day 4 post-induction, treated with DNase and qRT-PCR was performed as described previously (29–32), normalizing cDNA levels to tubulin. Primers amplifying nucleotides 301 to 407 of the MRB1590 open reading frame, Fwd (5′-TGTGAGATGCACATTTTTGT-3′) and Rev (5′-TGGTTTGTAGCACCTTCTCT-3′), were used to confirm MRB1590 RNA depletion.

### RT-PCR analysis of A6 transcripts

RNA was extracted from uninduced and tetracycline-induced MRB1590 RNAi cells. Oligo-dT primed cDNA was prepared by reverse transcription with Superscript III. A6-specific primers, 5′-GCGAATTCAAATAAGTATTTTGATATTATTAAAG-3′ and 5′-ATTAACTTATTTGATCTTATTCTATAACTCC-3′, which anneal to the never edited 5′ and 3′ regions that flank the edited part of the A6 mRNA, were used for RT-PCR analysis.

### Immunoprecipitation and western blot analysis

Immunoaffinity purification of MRB1590 was carried out using 2 × 10^10^ procyclic form cells containing the endogenous MRB1590-PTP construct. Expression of MRB1590-PTP was verified by western blot using affinity purified rabbit anti-protein C. Tandem affinity purification and nuclease treatment of cellular lysates was performed as described previously (22,33) using IgG Sepharose 6, fast flow chromatography and TEV protease cleavage. TEV cleavage samples were analyzed by western blot using polyclonal antibodies against Protein C, MRB3010 (22), MRB11870 (22), GAP1 (21), TbRGG2 (31), TbRGG1 (25) and MRB6070 (22).

### Expression and purification of *T. brucei* MRB1590

To obtain pure MRB1590 for structural and biochemical studies, the sequence encoding the full length *T. brucei* MRB1590 protein was cloned into the pMCSG7 expression vector using ligation-independent cloning (34). The vector was transformed into *E. coli* C41(DE3) cells. Protein expression was induced when the cells reached an *A*_600_ = 0.4–0.6 by the addition of 0.1 mM isopropyl β-d-1-thiogalactopyranoside for 4 h at 37°C. The protein was purified in one step using nickel-nitrilotriacetic acid column chromatography. Structure determination (see below) of full length MRB1590 revealed that its N-terminal region was largely disordered. Indeed, residues ∼1–30 are predicted to encode a mitochondrial targeting sequence, which are rich in basic and hydrophobic residues. Thus, different MRB1590 truncations were generated and cloned into pMCSG7. Studies showed that MRB1590(10–668) expressed optimally and was as active as the full length protein in RNA binding and ATPase activity assays. Hence MRB1590(10–668) was used for all studies unless otherwise stated.

### Crystallization and structure determination of MRB1590

Purified full length MRB1590 was concentrated to 5 mg/ml and crystallized via hanging drop vapor diffusion using 20% (w/v) PEG 3350, and 0.15–0.2 M sodium/potassium tartrate as a crystallization reagent. The structure was solved by single wavelength anomalous diffraction (SAD) using data collected from a crystal that had been soaked overnight with saturating concentrations of thimerosal. The heavy atom substructure was obtained using Phenix (34) and the MRB1590 structure traced into the density modified SAD electron density map using Coot. The structure contains one MRB1590 subunit in the crystallographic asymmetric unit (ASU) and a dimer is generated by crystallographic symmetry. The final model includes MRB1590 residues 52–174, 185–493, 505–525, 533–602 and 609–665, 1 ADP molecules,1 magnesium ion and 118 water molecules and has *R*_work_/*R*_free_ values of 20.4%/26.2% to 2.6 Å resolution. The structure of MRB1590 bound to AMP–PNP was obtained by mixing MRB1590(10–668) (in which ADP had been removed by EDTA treatment) with AMP-PNP and MgCl_2_ to final concentrations of 5 mM and 1 mM, respectively. This complex was then combined 1:1 with the crystallization reagent composed of 0.1 mM 1:2:2 dl-malic acid:MES [2-(*N*-morpholino)ethanesulfonic acid]:Tris (MMT) buffer, pH 9.0 and 20–25% (w/v) PEG 1500. The structure, which contains two subunits in the ASU, was solved by molecular replacement using Phaser (35). The structure was manually rebuilt in Coot and refined using Phenix (35,36). The final model has *R*_work_/*R*_free_ = 23.3/29.9% to 3.0 Å resolution and includes MRB1590 residues 52–174, 185–498, 505–525, 533–602 and 609–665 for two MRB1590 subunits, 2 AMP-PNP molecules, four magnesium ions and 20 water molecules. A structure of MRB1590(10–668) bound to ADP in the presence of poly-U_15_ was obtained by mixing a protein-ADP solution equimolar with polyU_15_ RNA and using 25% (w/v) PEG 3350, 0.2 M magnesium acetate as a crystallization solution. The structure was solved using Phaser (35). Although there was clear density for ADP, only sparse density was observed that could be attributed to the RNA near the N-domain pore region. The final structure contains MRB1590 residues 49–176, 183–495, 501–526, 533–665, 1 ADP molecule, 2 magnesium ions and 609 water molecules and has *R*_work_/*R*_free_ values of 16.8%/19.8% to 2.05 Å resolution All X-ray intensity data were collected at Advanced Photon Source beamline 22-BM and the data processed using HKL 2000 (37). The final crystallographic and refinement statistics for all the MRB1590 structures can be found in Table 1.

**Table 1.** Selected crystallographic statistic for MRB1590 structures

	MRB1590-ADP, crystal form 1	MRB1590–AMP–PNP complex	MRB1590-ADP, crystal form 2 (with poly-U)
Cell constants	*a* = 105.5 Å	*a* = 103.8 Å	*a* = *b* = 215.4 Å
	*b* = 184.7 Å	*b* = 71.5 Å	
	*c* = 73.6 Å	*c* = 103.5 Å	*c* = 100.1 Å
	*α* = *β* = *γ* = 90°	*α* = *γ* = 90°	*α* = *β* = *γ* = 90°
		*β* = 120°	
Space group	*C*222_1_	*P*2_1_	*I*422
Resolution (Å)	25.60–2.60	44.80–3.00	41.80–2.05
*R*_sym_ (%)^a^	8.7 (60.7)	11.0 (35.0)	6.5 (25.4)
Mean *I/*σI	31 (4.0)	9.3 (3.0)	24.5 (2.4)
Total reflections (#)	692 747	79 737	367 705
Unique reflections (#)	73 564	48 631	73 541
Refinement statistics			
Completeness (%)	100 (100)	96.6 (78.9)	100 (99.9)
Resolution (Å)	25.60–2.6	44.80–3.00	41.80–2.05
*R*_work_/*R*_free_ (%)^b^	20.4/26.2	23.3/29.9	16.8/19.8
RMSD			
Bond lengths (Å)	0.07	0.08	0.012
Bond angles (°)	1.0	1.2	1.59
Ramachandran			
Most favored region (%)	95.1	95.3	97.1

^a^*R*_sym_ = ΣΣ|*I_hkl_* - *I_hkl(j)|_*/Σ*I_hkl_*, where *I_hkl(j)_* is the observed intensity and *I_hkl_* is the final average value of intensity.

^b^*R*_work_ = Σ∥*F_obs_*|-|*F_calc_*∥/Σ|*F_obs_*| and *R*_free_ = Σ∥*F_obs_*| -|F*_calc_*∥/Σ|F*_obs_*|, where all reflections belong to the test set of 5% of the data randomly selected and not used in the atomic refinement.

Data for the high resolution shell are shown in parentheses.

### Fluorescence polarization (FP) experiments

FP experiments were performed using a PanVera Beacon 2000 FP System at 25 °C. 3′-Fluoresceinated oligonucleotides were utilized for RNA binding experiments. The oligos were heated to 99°C and slow cooled to room temperature prior to use to remove any secondary structure. RNA substrates that were measured include the GC-rich putative pause site in the A6 transcript, its complement, a double stranded (ds) GC-rich RNA, poly-U, poly-A and a dsRNA harboring the predicted hairpin in the A6 putative pause site. Regions of the RSP12, ND7 and COXII mRNA transcripts were also used in binding studies. For each experiment, MRB1590 was titrated into a 0.995 ml reaction cell containing a buffer of 20 mM Tris HCl, pH 7.5, 150 mM NaCl, 5% (v/v) glycerol, 1 mM MgCl_2_, 2 mM ADP or AMP–PNP with 1 nM fluoresceinated oligonucleotide. All FP experiments were fit to a bimolecular binding model by nonlinear regression. The MRB1590 concentration used to fit the data corresponds to that of the dimer. MRB1590 mutants, R199A–R200A, R525A–R526A–R527A–R529A, F96A, Y637A and R191A were generated with Quikchange and analyzed for RNA binding using FP as per the wild type protein.

### Characterization of nucleotide binding by MRB1590 using ITC

Isothermal titration calorimetry experiments were conducted with a MicroCal VP-ITC. Before ITC experiments, MRB1590 was treated with 0.1 mM EDTA for 1 h at 4°C to remove bound nucleotide. The protein was then dialyzed overnight into an ITC buffer consisting of 20 mM Tris–HCl, pH 7.5, 150 mM NaCl, 5 mM MgCl_2_, 5% (v/v) glycerol. ADP or AMP–PNP was dissolved in the same buffer. 28–30 aliquots of 10 μl of 1 mM nucleotide were titrated into 1.3 ml of MRB1590 (36 μM). The experiments were all done at 25°C.

### ATPase activity assays

The ATPase activity of MRB1590 was measured using a continuous, regenerative assay, as previously described (38). Briefly, the ATP hydrolysis rate is determined from the decrease in adsorption of NADH (extinction coefficient = 0.00622 μM^−1^ cm^−1^) at 340 nM, as measured using a PerkinElmer Lambda 25 UV-VIS Spectrometer. The assay buffer contained 20 mM Tris–HCl, pH 7.5, 150 mM NaCl, 5 mM MgCl_2_ and the reaction mixture included 0.5 mM phosphoenolpyruvate, 0.3 mM NADH, 20 units/ml pyruvate kinase, 20 units/ml lactate dehydrogenase, and 0.5 mM ATP. Hydrolysis was plotted against MRB1590 concentration and the slope of the line taken as the hydrolysis rate. This assay was carried out in the absence and presence of RNA.

### Size exclusion chromatography (SEC) analysis of MRB1590

For SEC, purified MRB1590 was concentrated to 5 mg/ml and applied to a Superdex 200 size exclusion column in a buffer containing 20 mM Tris–HCl, pH 7.5, 300 mM NaCl, 5% (v/v) glycerol. A single peak was obtained from the size exclusion column. The elution volume of MRB1590 was compared to a series of standard proteins to determine the molecular weight and hence oligomeric state of MRB1590.

## RESULTS

### *In vivo* characterization of MRB1590 and its role in kRNA editing

**Figure 1.**
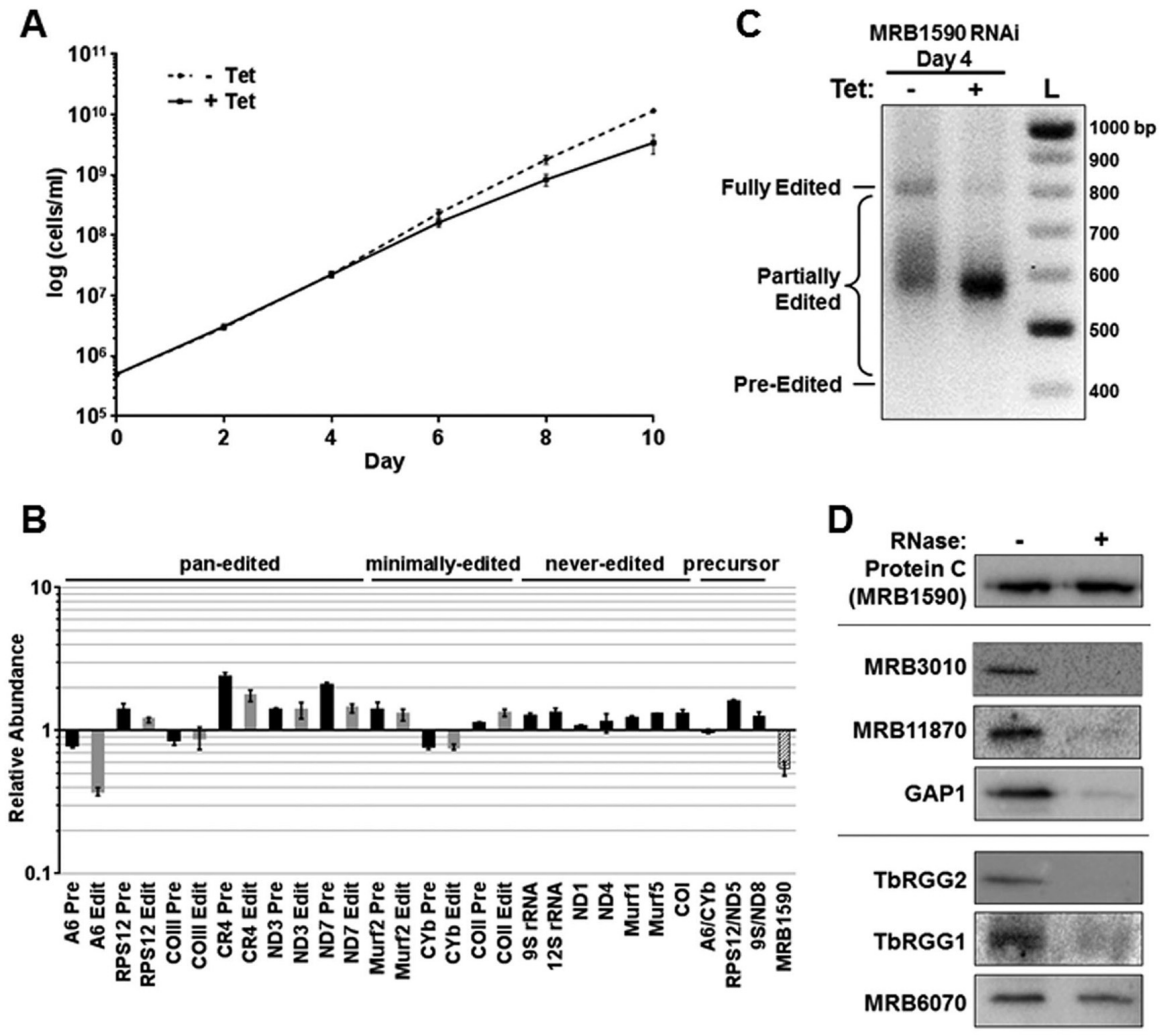
MRB1590 function and interactions *in vivo*. (**A**) Growth was monitored for 10 days in triplicate cultures of procyclic form MRB1590 RNAi cells in which MRB1590 downregulation was either uninduced (dotted line) or induced by addition of 2.5 μg/ml tetracycline (solid line). The data are presented as the average ± standard deviation of triplicate determinations. (**B**) RNA was isolated from procyclic form MRB1590 RNAi cells on day 4 post-induction with tetracycline. RNAs were quantified by qRT-PCR using primer sets specific for selected never-edited, pan-edited, minimally-edited, dicistronic precursor, and MRB1590 RNAs. Relative RNA abundance indicates RNA levels in tetracycline-induced cells compared to those in uninduced cells. RNA levels were standardized to tubulin (*n* = 5–13), and numbers represent the mean and standard error. (**C**) Agarose gel analysis of A6 RT-PCR reactions using RNAs isolated from MRB1590 RNAi cells that were grown in the absence or presence of tetracycline for 4 days. Reverse transcription was carried out with an oligo(dT) primer and PCR was done with primers specific to the 5′ and 3′ ends of the A6 mRNA to amplify the entire population of mRNAs including pre-edited, partially edited, and fully edited. (**D**) MRB1590-PTP and associated proteins were isolated by IgG Sepharose chromatography and TEV protease cleavage from untreated or RNase-treated extracts of cells expressing a C-terminal PTP-tagged MRB1590. TEV elutions were analyzed by western blot for MRB1 complex components using the antibodies indicated.

To assess the role of MRB1590 in kRNA editing, we generated procyclic form *T. brucei* cells in which MRB1590 was down-regulated by RNAi in a tetracycline-dependent manner. MRB1590 mRNA was depleted to ∼50% of the level in uninduced cells and its down-regulation caused a modest growth defect that became apparent by day 6 post-induction (Figure 1A and B). We next used qRT-PCR with primers specific for pre-edited and edited mitochondrial RNAs to assess the effect of MRB1590 down-regulation at the RNA level. These assays revealed that MRB1590 knockdown led to a significant, 40%, depletion in the levels of edited transcripts encoding subunit 6 of the ATP synthase (A6) compared to that in uninduced cells. These studies also revealed a slight (2-fold) enhancement in the levels of edited ND7 transcript as well as a 2-fold effect on the pre- and edited levels of the CR4 transcript (Figure 1B). MRB1590 down-regulation did not affect mRNA or rRNA stability or processing of polycistronic precursor RNAs, as evidenced by the absence of any significant change in the levels of never-edited or precursor RNAs (Figure 1B).

The A6 transcript appeared to be significantly affected by MRB1590 levels. Hence, to further probe the effect of MRB1590 down-regulation on the editing of this transcript we performed a full gene PCR assay (21,30). Primers to the static never-edited 5′ and 3′ regions of the pan-edited A6 transcript were used to amplify the A6 mRNA population, including pre-edited, fully edited, and partially edited RNAs. Because uridine insertion is at least ten times more prevalent than uridine deletion, increased editing is revealed in this assay as larger amplicons by gel electrophoresis. As shown in Figure 1C, MRB1590 down-regulation caused a loss of fully edited A6 mRNA but did not lead to a corresponding accumulation of pre-edited mRNA, consistent with the qRT-PCR results. Rather, MRB1590 down-regulation led to a build up of cDNAs corresponding to partially edited transcripts of ∼580 nts. These data indicate that MRB1590 does not affect the initiation of A6 mRNA editing, but appears to facilitate the 3′ to 5′ progression of editing through a specific region of the A6 mRNA. The A6 transcript is edited by the addition of 448 uridines and deletion of 28 uridines, resulting in a fully edited sequence of 821 nucleotides (39,40). Based on the edited A6 mRNA sequence, we estimated that A6 editing is paused in MRB1590-depleted cells within a GC-rich region located between nucleotides 209–229 in the pre-edited sequence (Supplementary Figure S1). Interestingly, guanine and cytosine nucleotides in this region are predicted to base pair and form secondary structures, suggesting one possible mechanism for how RNA editing might be impeded at this location (Supplementary Figure S2) (41). MRB1590 appears to enable editing past this site.

Because MRB1590 was first identified in association with the MRB1 complex, we next sought to clarify its interaction with this essential RNA editing complex. To this end, we generated cells expressing C-terminally PTP-tagged MRB1590 (33) and performed tandem affinity purification followed by western blotting with specific antibodies. In untreated cell extracts, MRB1590 was associated with the MRB1 complex, including components of both the MRB1 core (MRB3010, MRB11870, and GAP1) and the TbRGG2 subcomplex (Figure 1D). However, when extracts were RNase treated prior to MRB1590 purification, these interactions were lost, indicating that MRB1590 association with the MRB1 complex is mediated by RNA. Likewise, MRB1590 binding to TbRGG1, a mitochondrial RNA binding protein with which it was previously reported to interact (25), is also RNA-mediated. MRB6070 is a zinc finger containing protein that interacts with MRB1 through RNA linkers (22), although its role in mitochondrial RNA metabolism is unknown. Coimmunoprecipitation experiments indicated that MRB1590 and MRB6070 are able to associate in an RNA-independent manner. Whether they form part of a complex or subcomplex, however, is unclear. Collectively, our *in vivo* results indicate that MRB1590 significantly impacts the editing of the A6 transcript and, to a lesser extent, the ND7 and CR4 transcripts. The data also suggest that the association of MRB1590 with the MRB1 complex is RNA-mediated.

### Crystal structures of MRB1590 reveal ATPase fold

**Figure 2.**
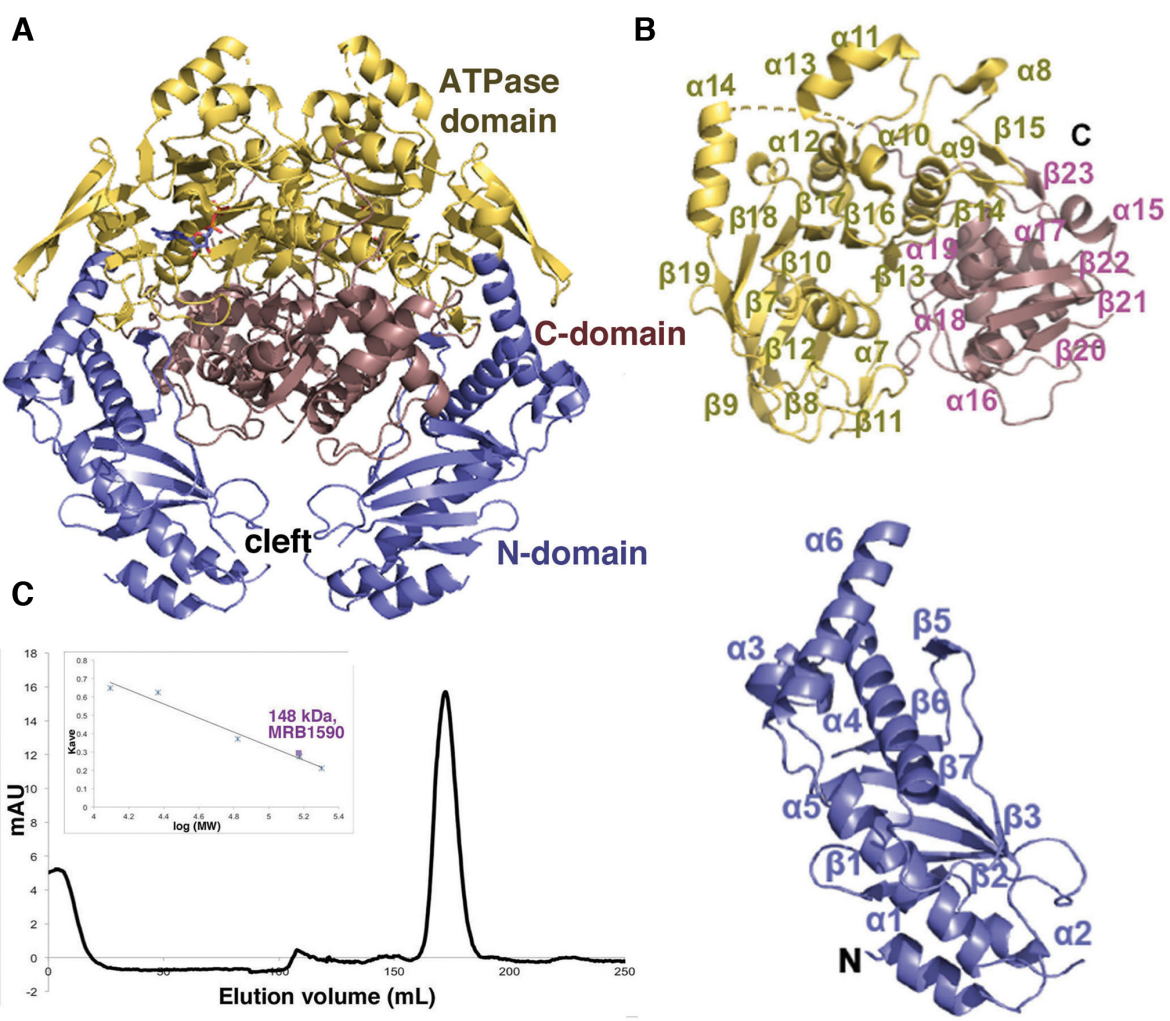
Structure of MRB1590. (**A**) Overall structure of MRB1590 with the N-domain, central ATPase domain and C-domain labeled and colored blue, yellow and pink, respectively. (**B**) Ribbon diagram showing the domains in (A) with secondary structural regions labeled. The N-terminal and C-terminal residues are also labeled. C) Size exclusion chromatography analyses of MRB1590. The protein runs as a dimer with a MW of 148 kDa (the monomer MW is 72 kDa). Shown is the elution profile and inset is the generated curve used to determine the MW, where the y axis is the elution volume normalized for column volume and the x axis is the log of the molecular weight (MW). The standards used for the analyses are cytochrome *c* (12.4 kDa), trypsin (23.3 kDa), albumin (66.4 kDa), alcohol dehydrogenase (150 kDa) and β-amylase (200 kDa).

The 72 kDa MRB1590 protein shows no significant sequence identity to any protein. Hence, to gain insight into its function at the molecular level, we determined its crystal structure by single wavelength anomalous diffraction (SAD). The structure was refined to final *R*_work_/*R*_free_ values of 20.4%/26.2% to 2.6 Å resolution (Table 1; Figure 2A and B; Materials and Methods). Although the full length protein was crystallized, N-terminal residues 1–51 were not visible. However, this region, which is not conserved among MRB1590 homologs (Supplementary Figure S3), includes the predicted mitochondrial targeting sequence and hence would be expected to be disordered. The MRB1590 structure revealed the presence of an expansive dimer that buries 2373 Å^2^ of subunit surface from solvent (42). Size exclusion chromatography (SEC) experiments supported that MRB1590 is a dimer (Figure 2C). The overall structure of MRB1590 can be divided into 3 main regions, the N-domain (residues 1–245), the central domain (residues 246–482) and the C-domain (residues 483–668) (Figure 2A-B). MRB1590 dimerization is mediated primarily by residues located in the central and C-domains of the protein and juxtaposes the two N-domains of the dimer together, creating a pore or cleft between them (Figure 2A).

The overall structure of MRB1590 appears unique. However, to ascertain if there are any structures or domains that show similarity to MRB1590 we performed structure homology searches with pdbfold/ssm (http://www.ebi.ac.uk/msd-srv/ssm/). These searches revealed that the central domain in MRB1590 (Figure 2A, yellow), displays strong structural similarity with ATPase proteins of the ABC class (43–47); root mean squared deviation (rmsd) = 2.9 Å for 291 corresponding Cα atoms of the MRB1590 dimer with that of the bacterial MJ0796 ABC transporter and rmsd = 2.6 Å for 158 corresponding Cα atoms in a comparison of the subunits of MRB1590 and the *E. coli* MalK ABC protein (Supplementary Figure S4A) (43,44). In fact, although no nucleotide was added to MRB1590 prior to crystallization, electron density for an ADP molecule was found in this central domain (Figure 2A). Unlike the ATPase domain, the MRB1590 N- and C-domains showed no structural similarity to any protein. Reduced stringency searches revealed weak similarity of the C-domain with the bacterial ArsR DNA binding, winged-HTH domain (rmsd = 3.4 Å for 67 corresponding Cα atoms between the MRB1590 C-domain and the ArsR DNA binding domain; Supplementary Figure S4B) (48). The N-domain of MRB1590 showed the strongest structural similarity with the chaperone ExsC from the *Pseudomonas aeruginosa* type II secretion complex, ExsC–ExsE (rmsd = 2.7 Å for 102 corresponding Cα atoms) (Supplementary Figure S4C) (49).

### Nucleotide binding by MRB1590

**Figure 3.**
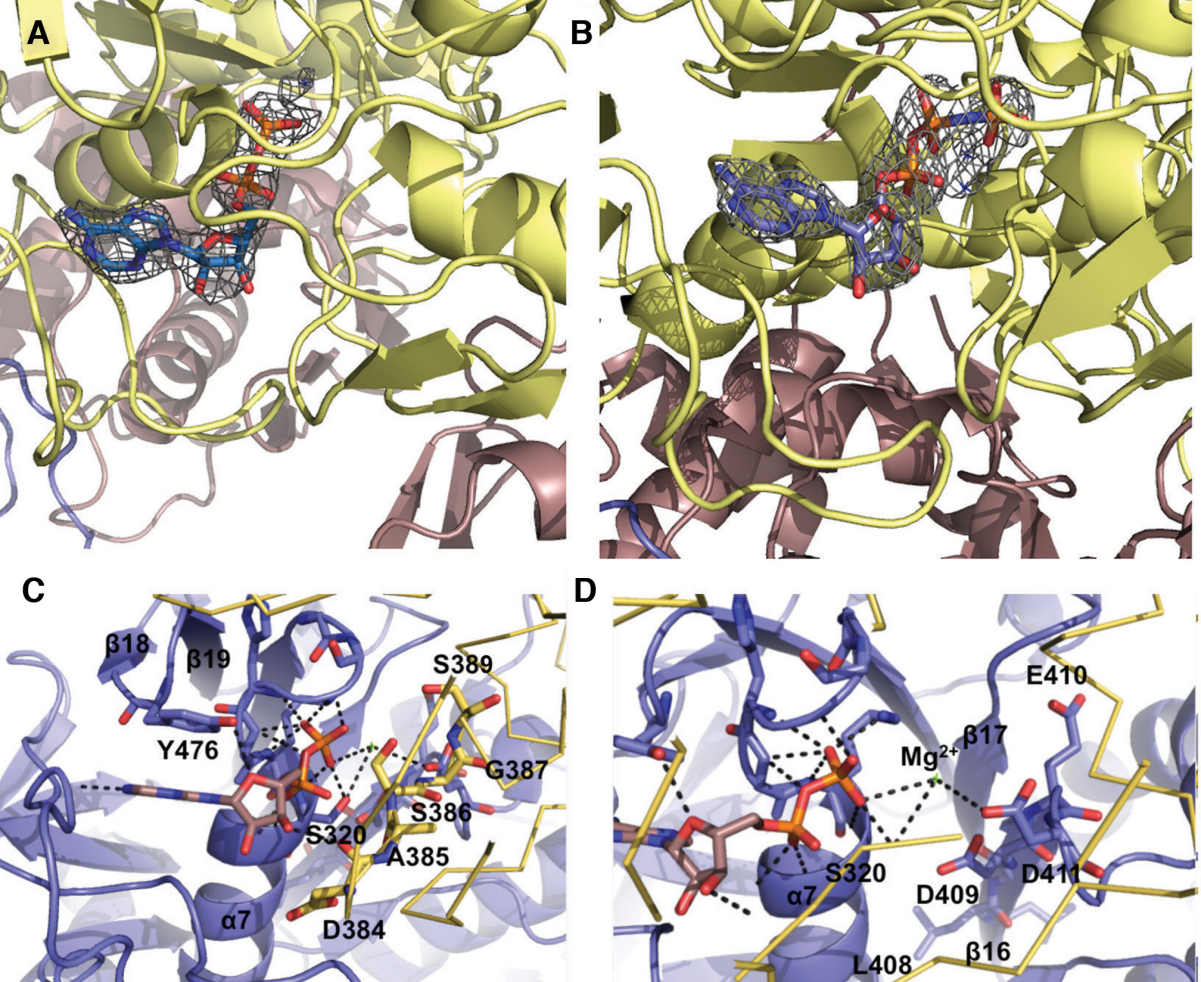
Nucleotide binding by MRB1590. (**A**) *F*_o_ − *F*_c_ omit map (blue mesh, contoured at 3 σ and calculated to 2.6 Å) in which the ADP was omitted from the refinement. Clear electron density for the ADP molecule bound near the P-loop region is observed. (**B**) *F*_o_ −*F*_c_ omit map (blue mesh, contoured at 3*σ* to 3.0 Å) showing density for the bound AMP–PNP, which was omitted from the refinement. (**C**) ADP binding by MRB1590. One subunit of the MRB1590 dimer is colored blue and the other is yellow. The specific amino acids in the Walker A or P-loop are highlighted as sticks. Also shown is the tyrosine residue, Tyr476, which stacks with the adenine base. The other subunit in the dimer, colored yellow, contributes the signature region, 385–389. The residues in this region are highlighted as yellow sticks and labeled. (**D**) Close up of the magnesium coordination site. The residues responsible for magnesium binding come from the Walker B motif, residues 407-411, which are located on β16. The magnesium ion is shown as a green cross and labeled.

The MRB1590 structure revealed that it contains an ABC-like ATPase fold embedded between two novel structural regions (45–47). As noted, although no nucleotide was provided in the crystallization setups, an ADP molecule was found in the structure (Figure 3A). These data suggest that MRB1590 function is likely tied to adenine nucleotide binding. Thus, to determine the affinity of MRB1590 for adenine nucleotides, we used EDTA treated protein (which removed any prebound magnesium complexed nucleotide) in isothermal titration calorimetry (ITC) experiments. These studies showed that MRB1590 bound ADP and the ATP analog AMP–PNP with *K*_d_s of 885 ± 49 and 1270 ± 210 nM, respectively (Supplementary Figure S5A and B). These binding affinities are comparable to those obtained for other ATPase ABC proteins (50).

To gain more insight into nucleotide binding by MRB1590, we obtained the structure of a MRB1590–AMP–PNP complex. Clear electron density was observed for the AMP–PNP molecule in this structure (Figure 3B). The MRB1590–AMP–PNP structure harbors the same overall subunit and dimer conformation as the ADP bound form and AMP–PNP binds in the same pocket as ADP. This binding site contains motifs consonant with MRB1590 belonging to the ABC class of Walker box ATPases. These proteins are defined by the presence of conserved structural regions called Walker A or P-loop and Walker B motifs, with consensus sequences, GxxxGxGK(S/T) and ΦΦΦΦD, respectively (47). MRB1590 contains a Walker A motif from residues 315–320 (GFHGKS) and a Walker B region from residues 407–411. As in typical ATPases, the MRB1590 Walker A motif is located in a strand-loop-helix. In the MRB1590 structures the phosphate moieties of the bound nucleotides are complexed by the backbone atoms of the Walker A motif while Asp411 from the Walker B motif interacts with a magnesium ion (Figure 3C and D). The magnesium ion is thought to help stabilize the formation of the transition state during ATP hydrolysis (45–47).

ATPase proteins typically form dimers or higher order oligomers as residues provided by the adjacent subunit in the oligomer are essential for nucleotide binding and hydrolysis by these proteins (45–47). In RecA-like ATPases, the adjacent subunit contributes an arginine finger, which inserts into the nucleotide binding pocket of the other subunit in the dimer and interacts with the nucleotide phosphate groups (51). Arginine fingers in this class of ATPases play the key role of facilitating ATP hydrolysis. In the case of ATPases of the ABC class, such as MRB1590, the arginine finger is replaced by a different motif, with the consensus, LSGGQ. In the MRB1590 structure, this signature motif corresponds to residues 385’ to 389’ (where prime indicates other subunit in the dimer), and contains the sequence, ASGGS (Figure 3C). In MRB1590, this signature motif contacts the AMP-PNP γ phosphate and hence, as in other ATPase ABC proteins, it is predicted to be important in ATP binding and hydrolysis. The structure of MRB1590 bound to AMP-PNP predicts that the small residues in the signature motif, in particular Ser386, would be essential for the close approach and binding of the γ phosphate moiety and that substitution of the serine to a larger residue would prevent AMP-PNP binding. To test this structure-based prediction, we mutated Ser386 to glutamate and measured adenine nucleotide binding by this mutant via ITC. These experiments showed that MRB1590(S386E) bound ADP with a similar affinity as the wild type, but was unable to bind AMP-PNP, consistent with our structural data (Supplementary Figure S5C). Taken together, these data indicate that MRB1590 binds nucleotides with similar affinity to other ATPase proteins and also reveals that its ASGGS signature is critical for ATP (AMP–PNP) binding.

### Characterization of MRB1590 as an RNA binding protein

Our *in vivo* data showing that MRB1590 effects kRNA editing and that its interaction with the MRB1 complex is RNA-meditated suggested the possibility that it might, itself, be an RNA binding protein. To test this hypothesis, we utilized fluorescence polarization (FP) and measured binding of MRB1590 in the presence of ADP to several RNA fragments. Poly-U sequences are found at the 3′-ends of gRNAs, hence we measured binding to a poly-U_15_ RNA. Also, as MRB1590 depletion correlated with altered editing of the A6 transcript and led to stalling at a certain GC-rich region, we also analyzed the ability of MRB1590 to bind a single stranded (ss) RNA fragment containing that site (5′-AGGCGGGCGGGCGACGGCGGU-3′). The FP experiments showed that MRB1590 bound these RNAs with *K*_d_s of 182 nM and 2.2 nM for the poly-U_15_ RNA and GC-rich sequence, respectively (Table 2). Stoichiometry experiments revealed a 1:2 ratio of RNA:MRB1590 subunit, indicating that the MRB1590 protein appears to be fully or nearly fully active in binding RNA as a dimer (Supplementary Figure S6). Also, as expected the K_d_ for the full length MRB1590, which contained the N-terminal mitochondrial targeting sequence, was the same as that of MRB1590(10–668) (Supplementary Table S1). We next measured MRB1590 binding to a ssA_15_ sequence, the ssRNA complement of the GC-rich sequence, a double stranded version of the GC-rich sequence (dsGC-rich) and the GC-rich hairpin that is predicted to form at the stalled region of the A6 transcript. MRB1590 showed no binding to the GC-rich hairpin and weak binding to the other RNAs (Table 2). The above experiments were all performed in the presence of ADP. Given the fact that the functions of ATPase proteins, such as MRB1590, are affected by binding different adenine nucleotides, we also carried out RNA binding experiments with MRB1590 in the presence of AMP-PNP. These studies showed that MRB1590–AMP–PNP bound RNA poorly compared to the complex with ADP (Table 2).

**Table 2.** MRB1590 RNA binding analysis

Substrate	*K*_d_ with ADP (nM)	*K*_d_ with ATP (nM)
U15 RNA	182 ± 30	471 ± 69
A15 RNA	437 ± 80	720 ± 27
GC-rich sequence RNA	2.2 ± 0.2	72 ± 8.9
Complement of GC-rich RNA	395 ± 95	377 ± 69
dsGC-rich RNA	50 ± 15	174 ± 20
GC-rich ‘hairpin’ RNA	N.B.	-

N.B. = no binding; - = not determined.

The first set of RNAs used to analyze MRB1590 RNA binding revealed a preference for binding to the ssGC-rich region from the predicted A6 pause site. However, these RNAs represented a limited set of sequences and hence we next examined binding to mitochondrial mRNAs regions within the RSP12, NAD7 (also called ND7) and COXII mRNA transcripts, which encode ribosomal protein subunit 12, subunit 7 of the NADH dehydrogenase complex and cytochrome C oxidase subunit II (Table 3). MRB1590-ADP bound these RNAs with ∼15–30 fold reduced affinity compared to the ssGC-rich site with the exception of region 2 of the NAD7 RNA (NAD7 #2), which showed only a ∼4-fold reduction in binding compared to the GC-rich ssRNA (Table 4). Notably, the NAD7#2 RNA contains two GCGA sequences, which is a motif also found in the A6 pause site.

**Table 3.** Mitochondrial mRNA substrate sequences

Mitochondrial mRNA	Sequence
COX II #1 (bps 484–512)	ACAAUAUCAAGUUUAGGUAUAAAGUAGA
COX II #2 (bps 513–543)	GAACCUGGUAGGTGTAATGAAAUAAUUUUGU
RSP12 #1 (bps 1–40)	CUAAUACACUUUUGAUAACAAACUAAAGUAAAAAGGCGAG
RSP12 #2 (bps 41–80)	GAUUUUUUGAGUGGGACUGGAGAGAAAGAGCCGUUCGAGC
NAD7 #1 (bps 1–32)	UGAUACAAAAAAACAUGACUACAUGAUAAGUA
NAD7 #2 (bps 217–248)	GAGAAGGCGAGGGCGACGGGCAAAAGAUUUU

**Table 4.** MRB1590 binding to selected mRNA transcript regions

Mitochondrial mRNA	*K*_d_ with ADP (nM)
COX II #1	44 ± 2.0
COX II #2	43 ± 1.6
RSP12 #1	63 ± 2.5
RSP12 #2	27 ± 2.8
NAD7 #1	50 ± 4.0
NAD7 #2	7.8 ± 0.5
40 mer GC-rich A6	1.3 ± 0.3

The NAD7#2 RNA was much longer than the ssGC-rich site tested. Hence, to determine if length plays a role in MRB1590 RNA binding affinity, we next measured the interaction of MRB1590 with a 40-mer RNA encompassing the GC-rich A6 pause site. MRB1590-ADP bound this RNA with a *K*_d_ of 1.3 nM, indicating that length does play a role in high affinity RNA interactions by MRB1590 (Table 4). This was confirmed in subsequent experiments, which showed that GC-rich RNAs 15 and 14 bp in length displayed significantly reduced binding by MRB1590 (Table 5). Taken together these data reveal MRB1590 binds RNA with high affinity in the presence of ADP and prefers GC-rich RNA sites, as found in the putative A6 pause site and ND7 transcript. These findings are particularly interestingly in light of our *in vivo* studies, which indicated that MRB1590 depletion affected the editing of the A6 and ND7 transcripts. Moreover, analysis of the CR4 pre-edited and edited sequences, which is also effected upon MRB1590 depletion, revealed a GCGA sequence in bps 235–238 of the pre-edited sequence that might be an MRB1590 target. Further studies will be needed to narrow down the binding specificity of the protein and its impact on editing of these transcripts.

**Table 5.** Length dependence on MRB1590–ADP RNA binding

Length of GC-rich RNA	*K*_d_ with ADP (nM)
21 mer	2.2 ± 0.2
18 mer	5.7 ± 0.2
16 mer	8.2 ± 0.6
15 mer	27.9 ± 9.1
14 mer	236 ± 4.1

### Structure of MRB1590-ADP in the presence of poly-U RNA reveals an open dimer conformation

**Figure 4.**
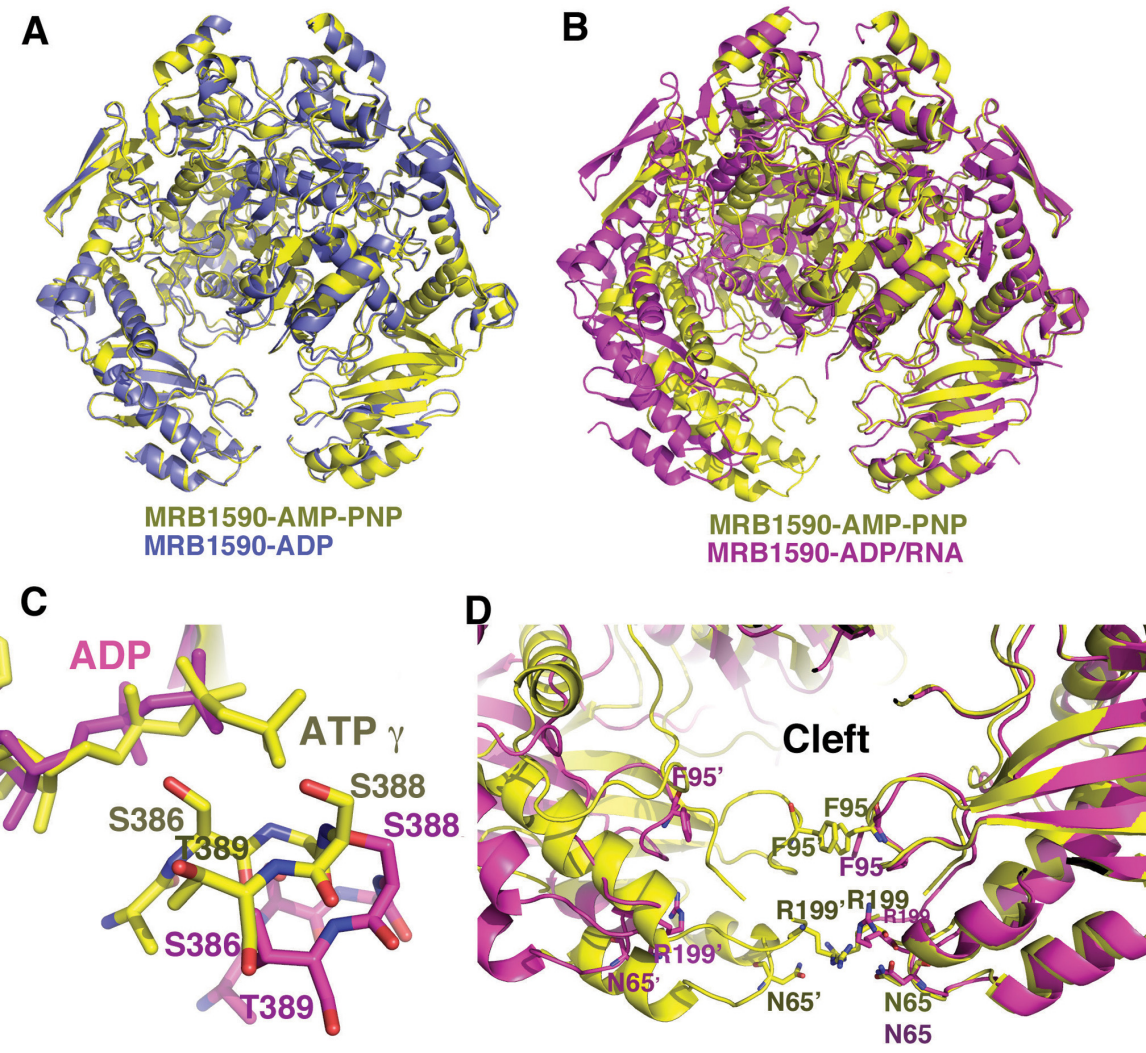
MRB1590 adopts open and closed states. (**A**) Overlay of the structures of MRB1590–ADP (blue) and MRB1590–AMP–PNP (yellow). The structures, including their dimer conformations are essentially identical. (**B**) Overlay of one subunit of the MRB1590–AMP–PNP (yellow) dimer onto that of the MRB1590–ADP complex obtained in the presence of RNA (magenta). Here, the subunits are the same but the dimers are dramatically different. The main result being that the N-domains are driven far apart in the RNA bound form, creating an open cleft between the N-domains. (**C**). Comparison of the location of the signature region in the non-overlaid subunit in the AMP–PNP bound or closed form (yellow) and the open (magenta) state. In the open state, the signature region is translocated too far to interact with the nucleotide that is bound in the other subunit. (**D**) Comparison of the N-domain cleft in the open and closed states. In the closed state, there are interactions between residues in the subunits of the dimer that block the channel. In particular, the 2-fold related Phe95 side chains and Arg199 side chains stack with each other. Arg199 also hydrogen bonds to Asn65’. These interactions are not present in the open state (magenta), which harbors a wide opening.

Our data indicate that MRB1590 binds RNA with high affinity in the presence of ADP. To try and gain insight into the molecular basis for the MRB1590–ADP interaction with RNA, we obtained a structure of MRB1590-ADP in the presence of poly-U_15_ RNA to 2.05 Å resolution (Table 1). Clear density for ADP was revealed in the structure, however, only weak electron density for the RNA was found near the MRB1590 N-domains. This likely reflects the fact that MRB1590 binds nonspecifically and with low affinity to this RNA. Strikingly, however, comparison of this structure to the previous MRB1590 structures revealed large conformational differences. Specifically, while the initial MRB1590–ADP and MRB1590–AMP–PNP structures largely adopt the same conformation (rmsd = 0.7 Å for 1158 corresponding Cα atoms) (Figure 4A), superimpositions of these structures with the MRB1590 structure solved in the presence of RNA showed that the latter structure has undergone a large shift in its oligomeric interface. This distinct dimer conformation appears to be the result of rotations originating at the nucleotide binding dimer interface (Figure 4B). As noted, the ASGGS motif mediates interaction with the ATP γ phosphate, which requires this specific dimer state. Upon ATP hydrolysis, this contact is lost allowing the MRB1590 dimer to rotate (Figure 4C). The MRB1590 structure solved with RNA buries less surface area (2127 Å^2^) than the other structures, due to its fewer cross dimer contacts.

The notable consequence of the structural changes found in the MRB1590 structure obtained in the presence of RNA is that the attached N-domains are significantly rotated relative to the rest of the protein. As a result the central cleft or pore located between the N-domains adopts an ‘open’ state. The average distance between N-domains in the RNA bound or open conformation is ∼28 Å compared to ∼3 Å in the closed state. In fact, there are direct contacts between residues in the N-domains in the closed state (Figure 4D). Specifically, the two-fold related Phe95 side chains stack together as do the side chains of Arg199. In addition, the Arg199 side chains make hydrogen bonds to Asn65’ (Figure 4D). Thus, the N-domain interactions largely block the N-domain pore in the closed form. By contrast, in the RNA bound structure there are no cross N-domain interactions and the pore is open. These data suggest that MRB1590 exists in an equilibrium between open and closed states, and that RNA binding stabilizes the open state (Figure 4D).

### Locating the RNA binding site of MRB1590

**Figure 5.**
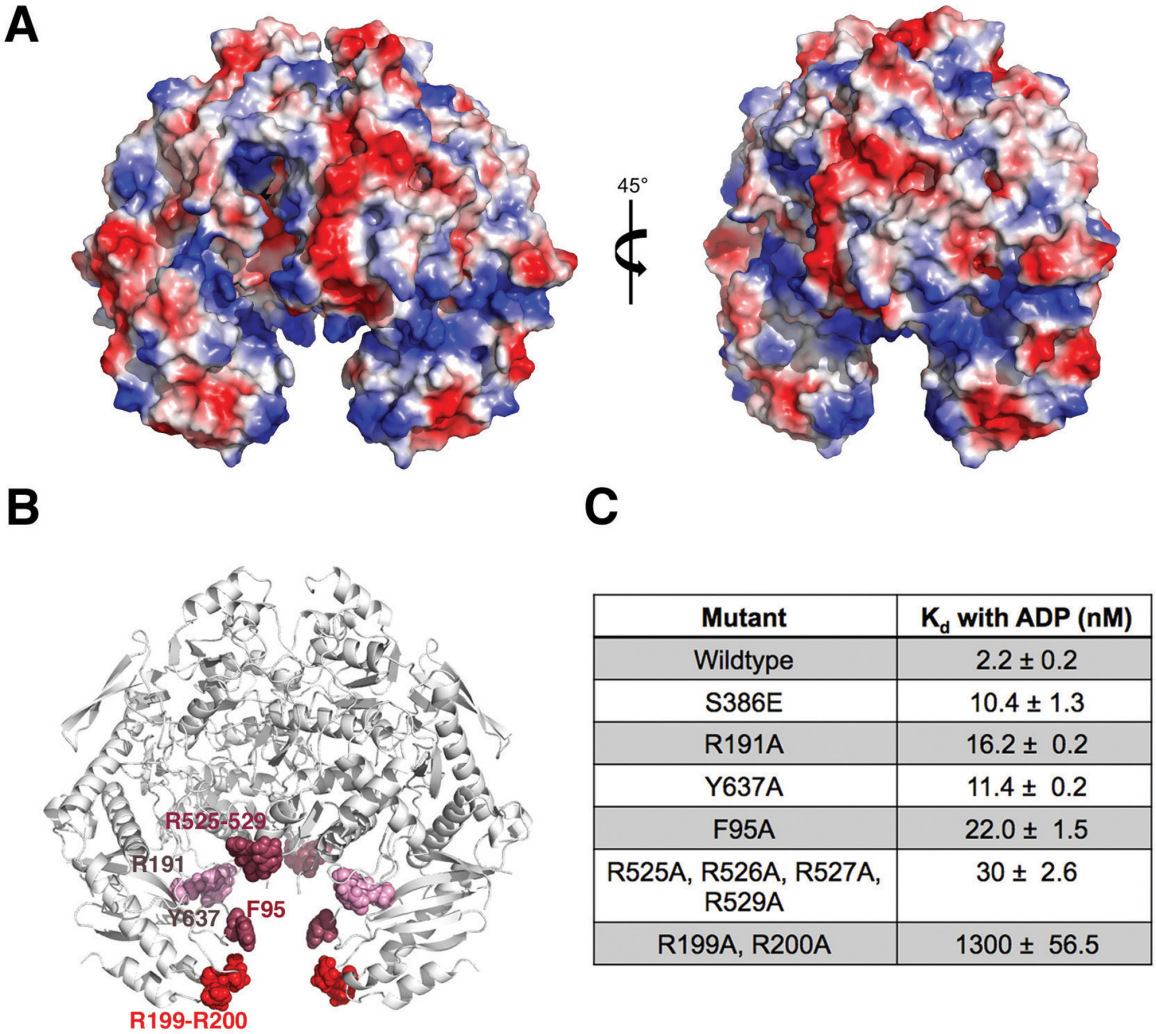
Electrostatic surface potential of MRB1590 and location of its RNA binding region. (**A**) Electrostatic surface representation of MRB1590, where blue and red represent positive and negative regions, respectively. The protein is shown in two orientations whereby the figure to the right shows the structure at a 45° rotation relative to the figure on the left. (**B**) Mapping the RNA binding region of MRB1590. Shown is a ribbon diagram of MRB1590 with the residues that were mutated to alanine shown and colored according to the impact of their mutation on RNA binding (labeled for one subunit). Mutations of residues that are colored red had a significant impact on RNA binding, residues in dark pink impacted RNA binding by 10-fold or more while residues colored light pink had little effect on RNA binding. (**C**) Table summarizing the *K*_d_s obtained for RNA binding by the MRB1590 mutants for theGC-rich sequence in the presence of ADP.

While MRB1590 contains no RNA interaction domains or motifs, our structure of MRB1590-ADP solved in the presence of RNA revealed spurious electron density near the N-domains as well as a distinct conformation in which the N-domains are splayed open. These data implicate the N-domain pore or cleft as the possible RNA binding region. Consistent with this hypothesis, the electrostatic surface potential of MRB1590 revealed strong basic patches within this cleft (Figure 5A). Examination of the MRB1590 amino acid sequence shows that these basic regions are particularly rich in arginine residues (Supplementary Figure S3). Arginines are known to provide binding specificity towards guanines and MRB1590 preferentially binds G-rich sequences, suggesting these as possible RNA binding residues. Particularly notable are the arginine patches, Arg525–Arg526–Arg527–Arg529 and Arg199–Arg200, which line the cleft. As noted, Arg199 lies directly in the center of the basic channel and in the closed state interacts with its dimer mate, Arg199’ to close the pore. Arg199 and Arg200 are completely conserved in MRB1590 homologs, while the Arg525–Arg526–Arg527–Arg529 patch is conserved in Trypanosome MRB1590 homologs (Supplementary Figure S3).

To determine if the MRB1590 arginine patches are involved in RNA binding, we mutated them to alanines and measured the binding of the resultant mutant proteins to the GC-rich sequence by FP. The R525A–R526–R527A–R529A mutant displayed a 14-fold reduction in binding compared to wt, while the R199A–R200A mutant decreased MRB1590 binding by 590-fold (Figure 5B-C). Mutation of the conserved basic residue, Arg191, found at the sides of the cleft had only a small effect on RNA binding. In addition to basic residues, aromatic amino acids have been shown to play key roles in protein interactions with RNA (52,53). Highly conserved aromatic residues in the MRB1590 basic cleft, Phe95 and Tyr637, were therefore chosen for additional mutagenesis studies. Like Arg199, Phe95 is located in the center of the cleft (Figure 4D). The Y637A mutation had little effect on RNA binding while the F95A showed a 10-fold decrease in binding. Thus, the combined data indicate that RNA binds in the basic cleft in the MRB1590 dimer. The completely conserved arginine patch composed of Arg199 and Arg200 appear critical for this binding (Figure 5B and C).

### MRB1590 ATPase activity

**Figure 6.**
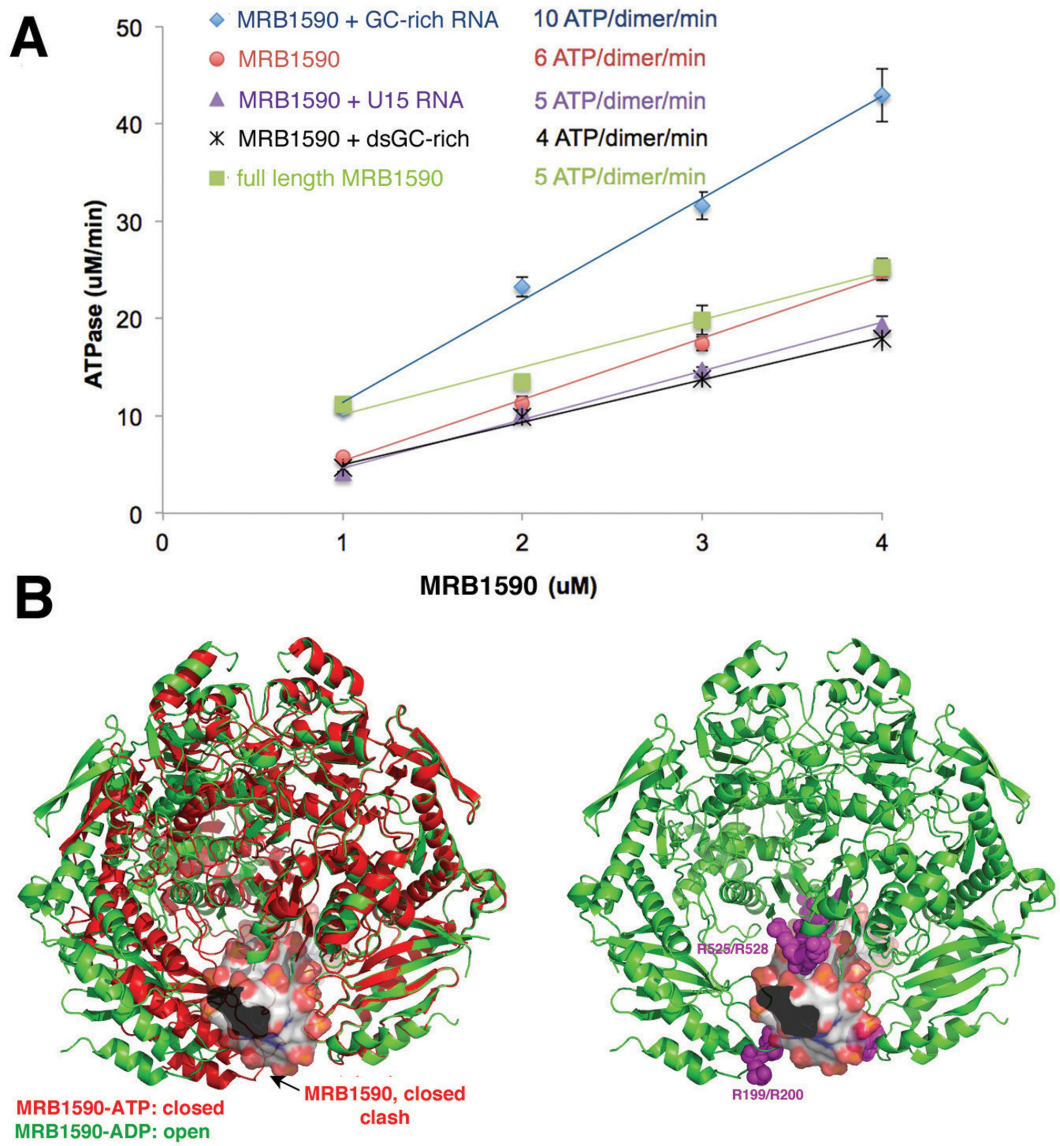
ATPase Activity of MRB1590 in the presence and absence of RNA. (**A**) Plot showing the ATPase activity for MRB1590 alone and in the presence of RNAs. MRB1590 has a low level of ATP hydrolysis in the absence of RNA and the rate increases slightly in the presence of the GC-rich sequence. (**B**) Left, overlay of one subunit of open (green) and closed (red) states of MRB1590 showing that the closed state cannot bind ssRNA (the ssRNA is modeled as surface representation) while the open state can. Right, mapping the mutations that had the largest affect on RNA binding on the MRB1590 open state.

Conformational changes are known to be employed by ATPases to drive the movement of substrates or even the unwinding of DNA or RNA (45). Thus, we utilized a NADH/ATP coupled assay to probe the ATPase activity of MRB1590. These experiments showed that MRB1590 hydrolyzes 6 ATP molecules/dimer/min (Figure 6A). We also measured MRB1590 ATPase activity in the presence of the poly-U_15_, the 21mer single stranded GC-rich sequence and dsRNA. In the presence of the dsRNA and the poly-U_15_ substrate the ATPase rate was unchanged while the addition of the GC-rich sequence resulted in a slight increase in the activity. Studies have shown that the ATPase activities of helicases are significantly affected in the presence of target, usually double stranded, RNA or DNA substrates (45). The finding that RNA molecules had little effect on MRB1590 ATPase activity suggests that it likely does not function as a helicase.

## DISCUSSION

MRB1590 was originally identified as a protein that is dynamically associated with the MRB1 complex. To gain insight into the function of the 72 kDa MRB1590 protein, we performed a battery of *in vivo*, structural and biochemical analyses. Structural studies showed that MRB1590 contains an ABC-like ATPase domain, which is embedded between N- and C-terminal domains that harbor novel folds. The MRB1590 ABC-like domain contains the signature motifs and residues found in related ATPase proteins and MRB1590 was shown to bind ADP and ATP at physiologically relevant concentrations. Moreover, crystal structures of MRB1590 in its ADP and AMP–PNP bound states showed that these nucleotides interact within the predicted Walker box regions of the MRB1590 protein. The crystal structures of MRB1590 contain a dimer with a significant buried surface area and SEC studies supported that MRB1590 is dimeric. The unique MRB1590 C-terminal domain provides key interactions for dimer formation. By contrast, the N-terminal arms, which make few dimer interactions, combine to create a basic pore or channel.

Our *in vivo* data indicated that MRB1590 is involved in the editing of the A6 transcript and also impacts editing of the ND7 and CR4 transcripts. These studies also showed that the interaction between MRB1590 and the MRB1 complex is RNA-meditated. These combined data suggested the possibility that MRB1590 may bind RNA. This was supported by FP studies. To gain insight into the MRB1590–RNA interaction, we obtained the structure of the protein in the presence of ADP and RNA. While the poly-U RNA utilized in crystallization, was largely disordered, this structure revealed a dramatically different conformation from the MRB1590 structures obtained in the absence of RNA. Specifically, the latter structures adopt a closed conformation in which the N-terminal arms are closely juxtaposed and make cross dimer contacts. Contrastingly, the structure obtained in the presence of RNA adopts an open conformation whereby the basic N-terminal arms are splayed apart.

The hypothesis that the N-terminal regions of MRB1590 mediate RNA binding was supported by mutagenesis studies, which revealed that several arginine residues in this channel, including Arg199, are important for high affinity RNA binding. Strikingly, our structures suggest that ATP binding would favor the closed state, in which the RNA binding cleft is largely blocked at the bottom edge by Phe95–Phe95’ and Arg199–Ag199’ stacking interactions (Figure 6B). Because these residues interact with each other in this closed state, they would not be available for contacting RNA (Figure 4D). Indeed, modeling shows that ssRNA can fit in the pore in the open state (Figure 6B). Thus, MRB1590 might act as a chaperone to bind and stabilize ssRNA to aid in kRNA editing processes. In this regard it is interesting that the RNA binding N-domain of MRB1590 shows structural similarity to chaperones (Supplementary Figure S4C). A chaperone might function at a stall or pause site, such as in the A6 transcript, by preventing formation of secondary structures that would be deleterious to the editing process. By contrast, the interaction of MRB1590 with a stably formed single stranded region could impede editing, which might explain the increase in editing of the ND7 transcript in MRB1590 depleted cells. The *in vivo* role of such inhibition is, however, unclear. Further, how MRB1590 might effect editing of both pre- and edited CR4 transcripts is currently not known. Interestingly, however, all the transcripts that were effected by MRB1590 depletion, A6, ND7 and CR4, share one or more GCGA motifs and binding studies showed that the protein interacted with RNA from the ND7 and A6 transcripts that contain these motifs. However, the binding appears complex; it appears both context and length specific. Clearly future studies will be needed to obtain a complete molecular description of the mechanism(s) of RNA binding by MRB1590 and its roles in kRNA editing.

## ACCESSION NUMBERS

MRB1590 coordinates and structure factors have been deposited in the Protein Data Bank under the Accession codes 4YJ1, 4YIX and 4YIY.

## Supplementary Material

SUPPLEMENTARY DATA
