# Supplementary material for: Structures of the T. brucei kRNA editing factor MRB1590 reveal unique RNA-binding pore motif contained within an ABC-ATPase fold
Source: Nucleic Acids Res. 2015 Jun 27;43(14):7096–109. doi: 10.1093/nar/gkv647 (PMC4538832; doi:10.1093/nar/gkv647)
Supplement: SUPPLEMENTARY DATA [file supp_43_14_7096__index.html]

Structures of the T. brucei kRNA editing factor MRB1590 reveal unique RNA-binding pore motif contained within an ABC-ATPase fold — Structures of the T. brucei kRNA editing factor MRB1590 reveal unique RNA-binding pore motif contained within an ABC-ATPase fold — SUPPLEMENTARY DATA 

# Structures of the *T. brucei* kRNA editing factor MRB1590 reveal unique RNA-binding pore motif contained within an ABC-ATPase fold

## SUPPLEMENTARY DATA

- SUPPLEMENTARY DATA
