# Supplementary material for: Structures of the T. brucei kRNA editing factor MRB1590 reveal unique RNA-binding pore motif contained within an ABC-ATPase fold
Source: Nucleic Acids Res. 2015 Jun 27;43(14):7096–109. doi: 10.1093/nar/gkv647 (PMC4538832; doi:10.1093/nar/gkv647)
Supplement: SUPPLEMENTARY DATA [file supp_gkv647_nar-00678-y-2015-File008.pdf]

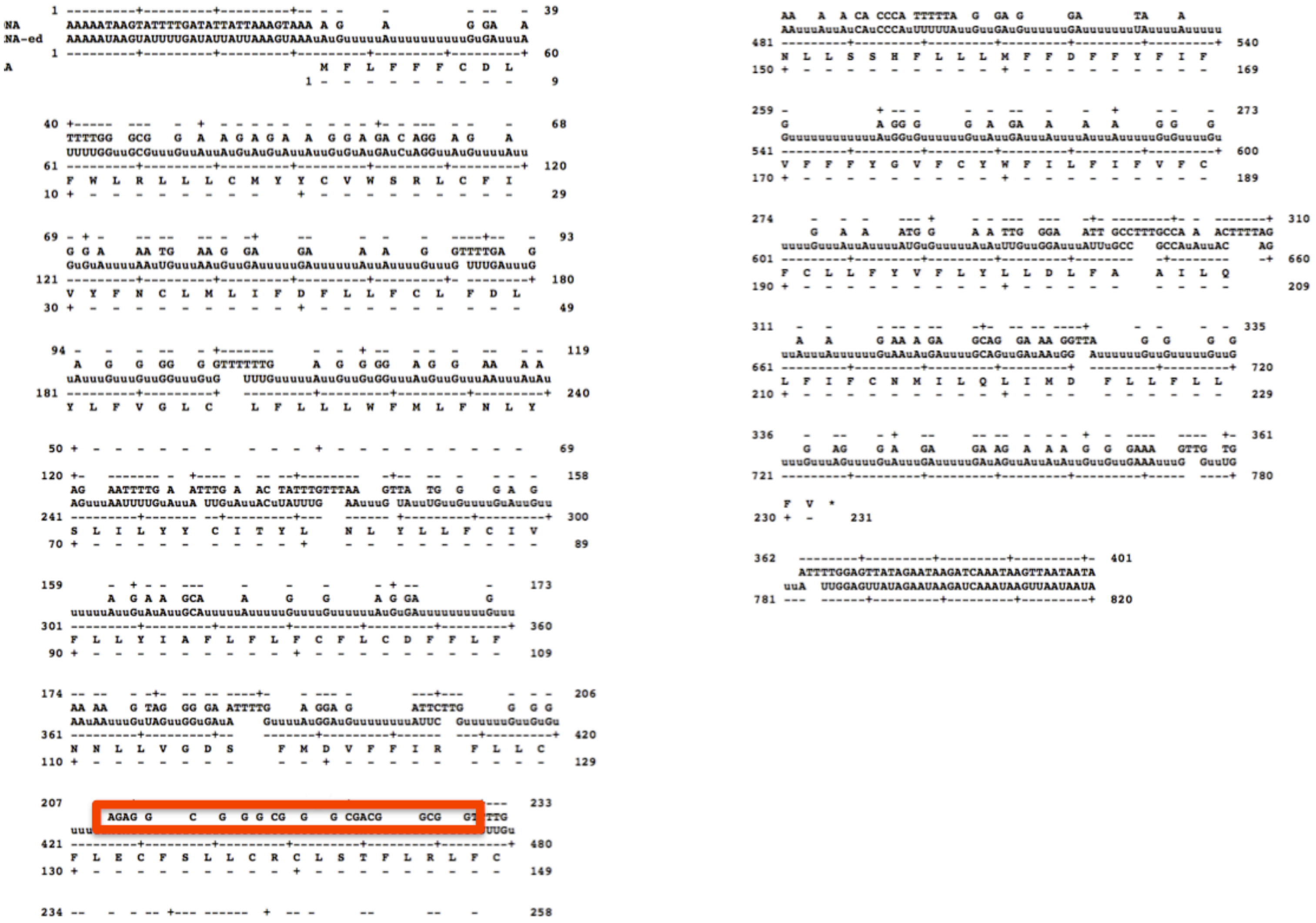

Figure S1. The *T. brucei* A6 non-edited (DNA) and edited (RNA-ed) sequences (originally from http://dna.kdna.ucla.edu/trypanosome/seqs/tba6ed.map.html). Also shown are the resultant amino acids (AA) that are encoded in the edited mRNA. The complete editing of this transcript requires the addition and deletion of 448 and 28 uridines, respectively. The region where editing appears to be paused in MRB1590 knock-downs is boxed in red.

```
AGAG-----|  GG
         GC   G
         CG   C
UGGCGGCAG^  GG
```

Figure S2. RNA secondary structure predicted to form in the putative pause region in the A6 transcript (Figure S1). Obtained from the Mfold web server for nucleic acid folding and hybridization (41).

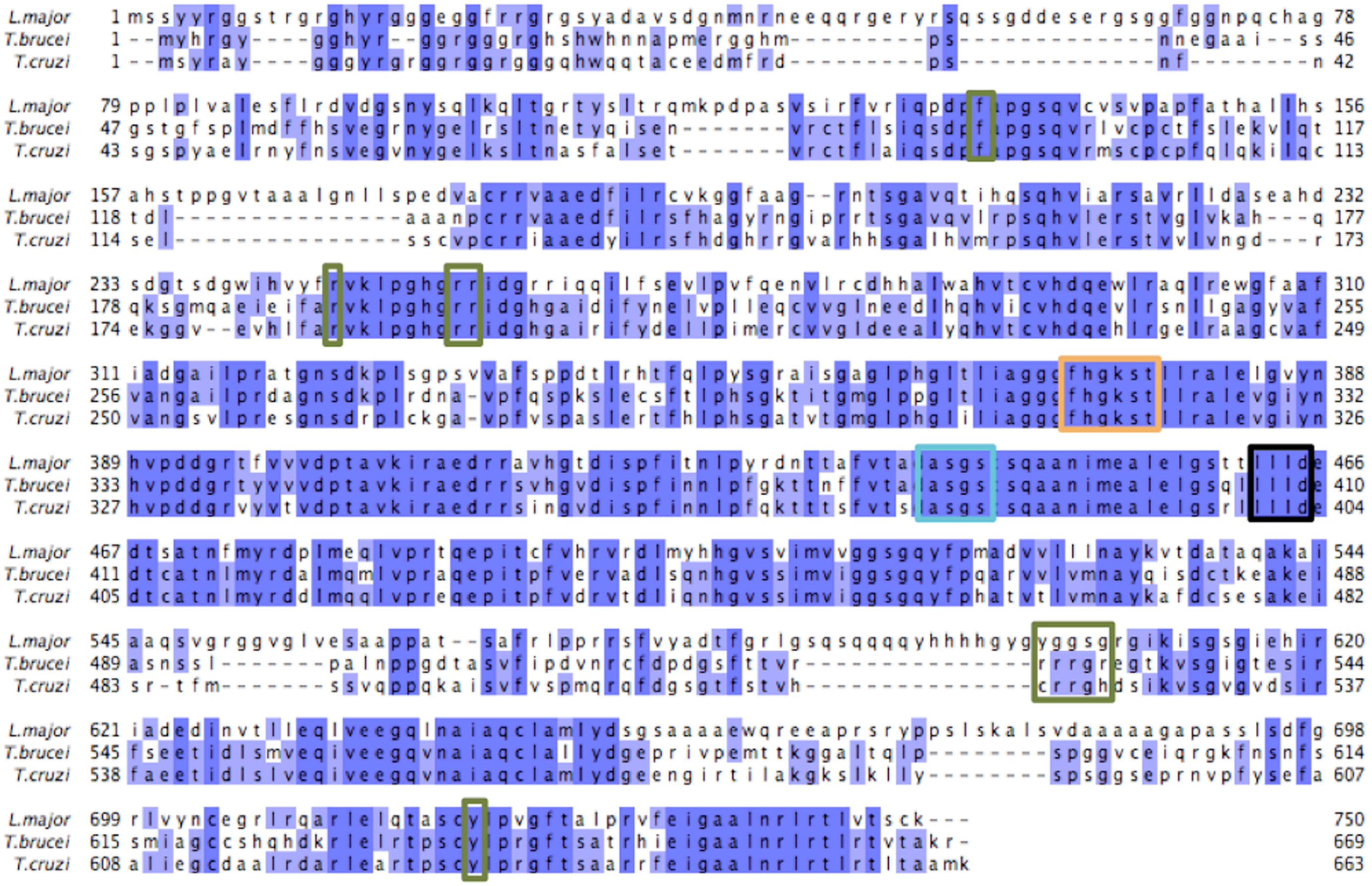

Figure S3. Sequence alignment of MRB1590 proteins from *Leishmania major*, *Trypanosoma cruzi*, and *Trypanosoma brucei*. The sequence homology is noted by the shade of blue highlighting; the darker the shade, the more homologous that residue is across all three species. The ATPase motifs are conserved in all MRB1590 proteins and are marked as follows, the Walker A motif is highlighted with an orange box, the Walker B motif with a black box, and the signature motif is highlighted with a cyan box. The residues mutated to test for RNA binding are highlighted with green boxes.

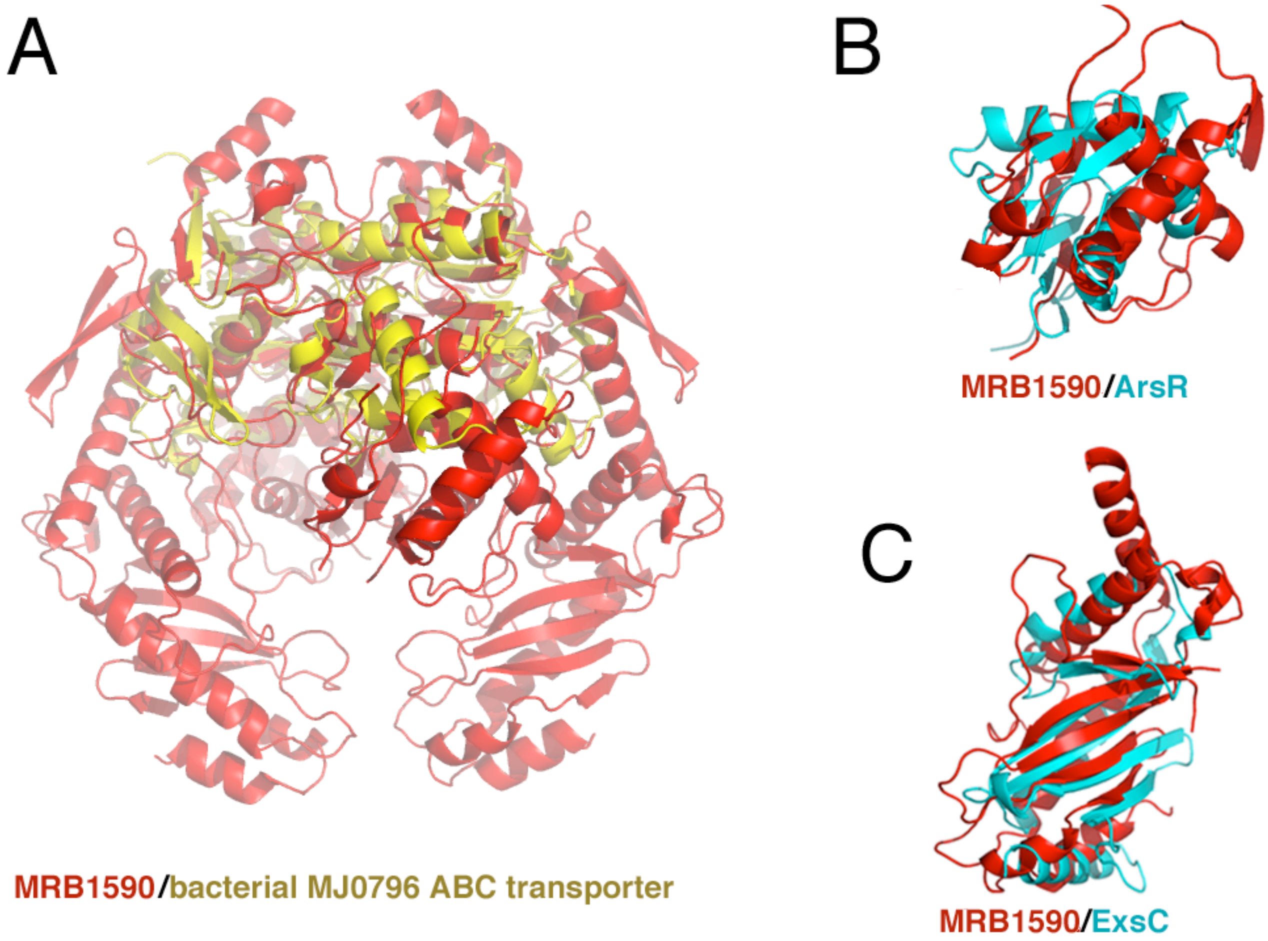

Figure S4. Results of structural similarity searches of MRB1590. A) Overlay of MRB1590 with the bacterial MJ0796 ABC transporter. MRB1590 is colored red and the bacterial MJ0796 ABC transporter is colored yellow. The ATPase domain of MRB1590 (residues 246-482) is homologous to this bacterial ABC transporter with an rmsd of 2.9 Å for 291 corresponding Cα atoms. B) Overlay of C-domain of MRB1590 (red) with AsrR DNA binding domain (cyan). C) Overlay of the N-domain of MRB1590 (red) with EsxC (cyan), a chaperone from the *Pseudomonas aeruginosa* type II secretion complex.

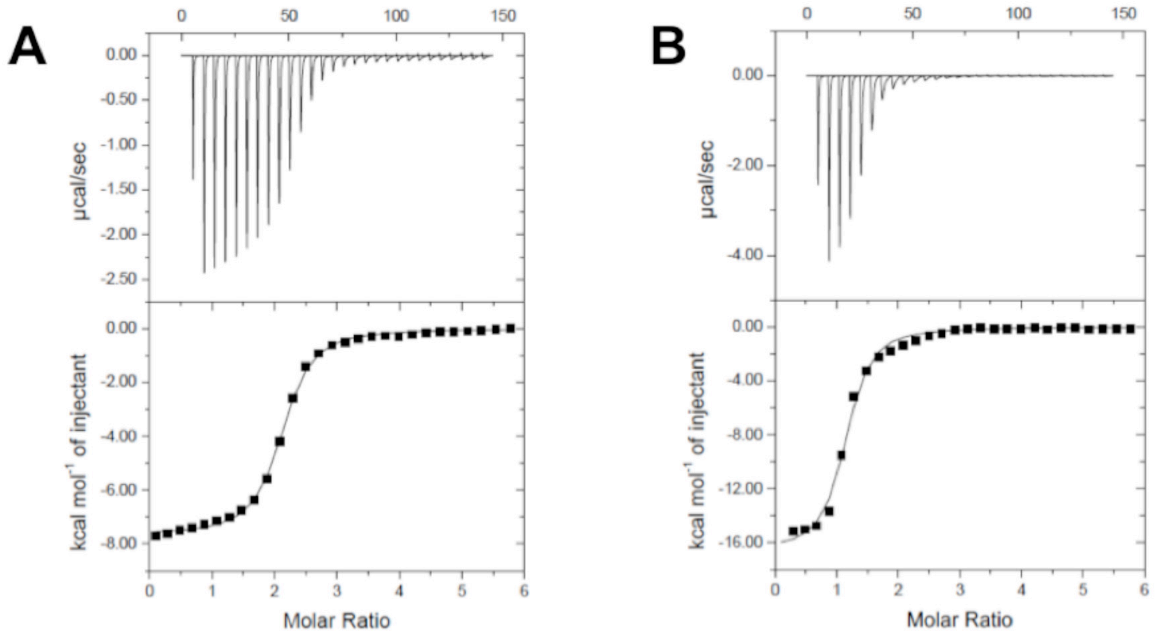

C

Protein	K_d ADP (nM)	K_d AMPPNP(nM)
Wild-type	885 ± 44	1270 ± 210
S386E	207 ± 60	N.B.

Figure S5. ITC analyses of nucleotide binding by MRB1590. A) ITC isotherm of ADP binding to MRB1590. B) Binding isotherm of the MRB1590-AMP-PNP interaction. C) Binding affinities of nucleotides to MRB1590 obtained from the ITC analyses. Also shown are the K_ds for MRB1590(S386E) binding to ADP and AMP-PNP.

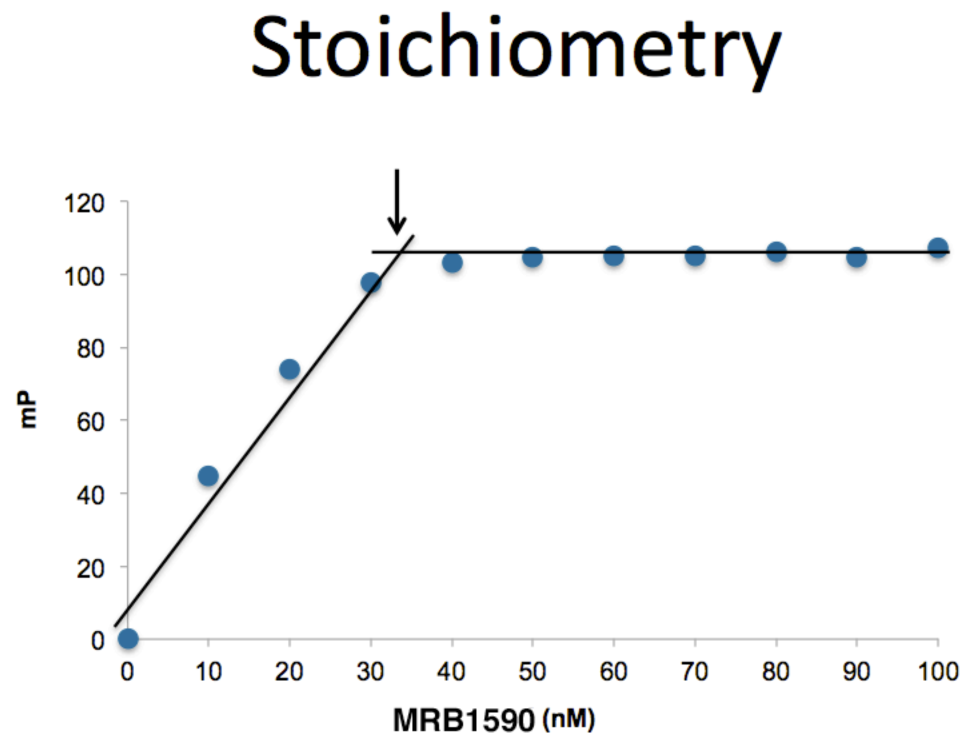

Figure S6. MRB1590 activity assay. The binding stoichiometry of MRB1590-ADP to the high affinity GC-rich site was determined using FP. For these experiments, the binding buffer and conditions were identical to those used in the binding affinity determination experiments except that the concentration of the RNA was increased to 20-fold higher than the K_d, thereby ensuring stoichiometric binding. The graph of the resulting data shows a linear increase in the observed millipolarization until saturation of the RNA, after which the curve levels off. The inflection point occurs at a MRB1590 monomer concentration of 40 nM, which, when divided by the concentration of cognate RNA (20 nM), indicates a stoichiometry off two MRB1590 subunits, or one MRB1590 dimer per RNA site.

Table S1: FL and Δ10 binding to GC-rich RNA

MRB1590	K_d with ADP (nM)
Full length	1.9 ± 0.2
Δ10	2.2 ± 0.2
